# How Does Income Heterogeneity Affect Future Perspectives on Food Consumption? Empirical Evidence from Urban China

**DOI:** 10.3390/foods11172597

**Published:** 2022-08-26

**Authors:** Wenbo Zhu, Yongfu Chen, Xinru Han, Jinshang Wen, Guojing Li, Yadong Yang, Zixuan Liu

**Affiliations:** 1 Rural Development Institute, Chinese Academy of Social Sciences, Beijing 100732, China; 2 College of Economics and Management, China Agricultural University, Beijing 100083, China; 3 Institute of Agricultural Economics and Development, Chinese Academy of Agricultural Sciences, Beijing 100081, China; 4 Institute of Agricultural Resources and Regional Planning, Chinese Academy of Agricultural Sciences, Beijing 100081, China

**Keywords:** food consumption, income heterogeneity, future perspective, China, EASI demand system model, dynamic simulation

## Abstract

China is undergoing a rapid dietary transition as well as a changing income distribution. In this paper, we examine the impacts of income heterogeneity on the prediction of food consumption using a dataset that covered 22,210 urban households in China’s 6 provinces. The two-stage Exact Affine Stone Index Implicit Marshallian Demand System (EASI demand system) model, which deals with the problem of censoring and endogeneity, is applied to estimate demand elasticity across income strata. Additionally, a dynamic simulation method considering income heterogeneity is conducted to predict future food consumption trends. The results reveal that income elasticity follows a decreasing trend with income growth. Furthermore, the results show that the consumption of major food items in the 15th period will increase by 7.9% to 42.0% over the base period. The growth potential of low-income groups is significantly higher than that of middle- and high-income groups. However, the prediction results may be overestimated if the differences in consumer behavior across income groups and the dynamic simulation procedure are not taken into account. Our study indicates that the consumption features of different income groups need to be included in food consumption forecasts. Moreover, the government should formulate food policies for different income groups to promote a sustainable food system transformation.

## 1. Introduction

The traditional food consumption structure in China that was centered on fiber-dominated foods has gradually shifted to a Western style of food consumption structure centered on animal food sources [1,2,3]. According to the National Bureau of Statistics of China (NBSC), in the last 20 years, the per capita consumption of grains and vegetables has dropped by 39.0% and 10.2%, respectively. Meanwhile, the per capita consumption of pork, beef and mutton, poultry, eggs, seafood, and milk increased by 7.9%, 121.1%, 244.1%, 86.3%, 109.0%, and 166.6%, respectively. The changing dietary patterns in China can be attributed to the fast economic growth which is accompanied by the rapid development of urbanization [4,5,6]. From 2001 to 2021, the per capita disposable income of Chinese residents increased from RMB 6824 to RMB 35,128, with a real growth rate of 6.1% annually, while the urbanization rate has increased from 36.2% to 64.7%. During the same period, the per capita Chinese food expenditure increased from RMB 1270 to RMB 7178, with a real growth rate of 15.0% annually.

However, changes in dietary structure may create potential health problems and environmental crises [7,8]. Excessive intake of fats and animal foods has led to over half of Chinese adults being overweight or obese, which may further increase the morbidity and mortality of chronic diseases and increase medical costs [9,10]. Additionally, dietary changes will increase greenhouse gas emissions and water demand, exacerbating climate change and putting significant pressure on resource management [11,12,13]. According to the FAOSTAT, China had the most GHG emissions (1.89 Gt CO_2_ eq yr^−1^) in terms of food system emissions in 2019, increased by 39.1% compared with the emission level in 2000 [14]. Furthermore, due to China’s large population size, small changes in food consumption structure will cause dramatic fluctuations in total food demand [6,15].

It is vital to accurately predict the trends of future diets in China. These projections enable policy-makers to identify the future health, nutritional, and environmental impacts of projected dietary changes [16]. Accurate predictions on diet trends can provide helpful information for national and international organizations involved in designing food policy [17,18]. Demand elasticity analysis indicates that food consumption responds to changes in income or other determinants, and the estimated demand elasticity can be used to predict the trends of food consumption [5,19]. This method has been widely used in food consumption prediction, but much uncertainty still exists [4,20,21]. The uncertainty of elasticity prediction mainly comes from the estimation of elasticity value. Chen et al. (2016) conducted a meta-analysis of the demand elasticity of 19 types of food in 85 pieces of literature on food consumption in China [22]. Among them, there are significant differences between food items and items inside the same food item. The variations in estimated demand elasticities can be attributed to different models, data, and estimation methods [5]. Therefore, more robust prediction methods and datasets are needed to ensure the accuracy and robustness of the prediction results.

Income is a crucial factor affecting food consumption change [5,23]. Engel’s and Bennett’s laws demonstrate the impact of income changes on food consumption [4,24]. There is evidence that the dietary structure of Chinese citizens will continue to change with the growth of income in the future, but the exact routes of these changes are still unclear [25,26]. There is heterogeneity in the preferences and consumer behavior of different income groups. Different income groups have different income levels, income growth patterns, and sensitivities to food prices, which is directly reflected in the different elasticities of demand across different income strata [2,27]. This means that using the average elasticity of the entire population to carry out food consumption forecasts may lead to biased results. Therefore, it is very meaningful to conduct a simulation for capturing the possible food consumption trends with more accurate information on elasticities across detailed income strata.

The purpose of this paper is to analyze the impact of income heterogeneity on future perspectives on food consumption. Specifically, this paper estimates food demand elasticities across different income groups in urban China using the EASI demand system model and analyzes the response of food consumption to income changes. Then, this paper predicts the future trends of food consumption in distinct scenarios using a dynamic simulation approach with segment income groups.

Two main contributions are made in this article to the literature. First, this paper uses the more advantageous EASI demand system model and microdata with significant sample sizes to estimate the food demand elasticity of different income groups. This study provides a quantitative basis for analyzing the characteristics of food consumption behavior across income groups. The study also provides parameter support for developing other food-consumption-related research. Second, this paper improves the application of the prediction method in the field of food consumption. The method fully considers the heterogeneity and the dynamic mechanism across income groups and corrects the estimation bias caused by the use of average population elasticity and static simulation. This research provides implications for the formulation of relevant food policies.

The structure of this paper is as follows. Section 2 presents the methodology and the data. In Section 3, the empirical results and simulation results are presented. Section 4 is the discussion, and Section 5 is the conclusion.

## 2. Materials and Methods

### 2.1. Study Design

There are two steps to conducting the analysis in this study. The first step is to estimate the income elasticity and price elasticity under the conditions of no income group, five income groups, and one hundred income groups using the two-stage EASI demand system model. The level of detail of the division is considered in the income groups. The second step is to use the dynamic prediction method embedded in income group differences to simulate the changing trend of food consumption. In the prediction process, the differential elasticity information of different income groups is used, and the static simulation mechanism is optimized into a dynamic process.

### 2.2. Two-Stage EASI Demand System Model

Assuming that preferences are weakly separable, a two-stage budgeting procedure is used to characterize consumer preferences for foods in urban China [19]. Consumers allocate their total expenditures between food and other goods and services in the first stage. In the second stage, consumers allocate their expenditures to thirteen food categories. Finally, the income elasticity and unconditional price elasticity are calculated by the parameters estimated at each stage [28].

#### 2.2.1. First Stage: Engel Model

In the first stage, this study adopts the Engel model [21,29,30]. Following the previous work, this study uses the extended Engel model, in which the core explanatory variables include the logarithm of total expenditure, the square of the logarithm of total expenditure, and the food price index. The model also controls for other commodity prices and demographic variables [31,32].

(1) $$W_{f}=\alpha_{0}+\beta_{1}lnX+\beta_{2}{\left(lnX\right)}^{2}+\beta_{3}lnP_{food}+\beta_{4}lnP_{other}+\overset{n}{\underset{k=1}{\sum }}\lambda_{k}Z_{k}+\mu \;$$

where $W_{f}$ represents the proportion of the total expenditure on the thirteen categories of food defined in this study in the total household expenditure; lnX is the natural logarithm of the total household expenditure; ${\left(lnX\right)}^{2}$ represents the square of the logarithm of the total household expenditure; $Z_{k}$ represents the kth demographic variable; μ represents the random disturbance term; $\alpha_{0},\;\beta_{1},\;\beta_{2},\;\beta_{3},\;\beta_{4},$ and $\lambda_{k}$ are all parameters to be estimated; and $lnP_{food}$ and $lnP_{other}$ represent the food price index and the price of other commodities, respectively, represented by the Stone price index.

#### 2.2.2. Second Stage: EASI Demand System Model

In the second stage, the EASI demand system model was applied, which can effectively capture the effect of income and price on food consumption. The EASI model not only shares all of the desirable properties of the Quadratic Almost Ideal Demand System (QUAIDS) model but also provides additional benefits. First, it is not subject to the three rank limitations of Gorman and allows the Engel curves to take arbitrary shapes [33]. Second, the EASI error term can be interpreted as unobserved consumer heterogeneity, while the QUAIDS residual does not have this interpretation. Third, the EASI model adds the interaction terms of total expenditure, price, and demographic variables, which have certain advantages and are extensible [34]. The EASI model has recently been widely used in food consumption research [6,35,36].

The EASI model is derived from the cost function based on consumer behavior theory. Consumers with observable characteristics (vector z) and unobservable characteristics (vector **ε**), given a commodity price (vector p), choose the proportion of budgeted expenditures *w* for each type of food to achieve a certain level of utility *u* so that the total cost C(p,u,z,ε) is minimized. According to Lewbel and Pendakur, the natural logarithm of the cost function C(p,u,z,ε) of the EASI model can be expressed as

(2) $$lnC\left(p,u,z,\varepsilon \right)=u+{\left(lnp\right)}^{\prime }\left[\left(\overset{R}{\underset{r=0}{\sum }}\alpha_{r}u^{r}\right)+Cz+Dzu\right]+\frac{1}{2}\overset{L}{\underset{l=0}{\sum }}z_{l}{\left(lnp\right)}^{\prime }A_{l}\left(lnp\right)+\frac{1}{2}{\left(lnp\right)}^{\prime }B\left(lnp\right)u+{\left(lnp\right)}^{\prime }\varepsilon \;$$

where $lnp={\left(lnp_{1},\ldots ,lnp_{J}\right)}^{\prime }$ represents the *J*-order vector of the logarithm of food prices; $z={\left(z_{1},\ldots ,z_{l}\right)}^{\prime }$ represents the *L*-order observable influence on consumer preference; $\varepsilon ={\left(\varepsilon_{1},\ldots ,\varepsilon_{J}\right)}^{\prime }$ represents the unobservable features of the *J*-order affecting consumption preferences and satisfies $1_{I}^{\prime }\varepsilon =0$; $1_{I}$ is a *J*-order column vector with all elements equal to 1; *u* is the utility level; $\alpha_{r}$ is a *J*-order parameter column vector satisfying $1_{J}^{\prime }\alpha_{0}=1$ and $1_{J}^{\prime }\alpha_{r}=0$ (*r* ≠0); *R* represents the rank of the demand system, which is an integer and 1 ≤ *R* ≤ *J* − 2; *J* is the total number of commodities in the demand system; $A_{l}$(*l* = 1,…, *L*) and B are both parametric symmetric matrices of order *J × J*, satisfying $1_{J}^{\prime }A_{l}=1_{J}^{\prime }B=0_{J}^{\prime }$ and when *l* = 0, $z_{0}$ = 1; and both C and D are *J × L* order parameter matrices, satisfying $1_{J}^{\prime }C=1_{J}^{\prime }D=0_{L}^{\prime }$. 

The Marshallian demand share (vector w) can be expressed as:

(3) $$w=\left(\overset{R}{\underset{r=0}{\sum }}\alpha_{r}y^{r}\right)+Cz+Dzy+\overset{L}{\underset{l=0}{\sum }}z_{l}A_{l}\left(lnp\right)+B\left(lnp\right)y+\varepsilon$$

(4) $$y=\frac{lnx-{\left(lnp\right)}^{\prime }w+\frac{1}{2}\sum_{l=0}^{L}z_{l}{\left(lnp\right)}^{\prime }A_{l}\left(lnp\right)}{1-\frac{1}{2}{\left(lnp\right)}^{\prime }B\left(lnp\right)}$$

This *y* has many of the properties of log real expenditures. It equals a cardinalization of utility *u*, it is affine in nominal expenditures *x*, and it equals *x* in the base period when all prices equal one. Like any money-metric utility measure, *y* is just a mathematically convenient representation of utility. Therefore, the demand system composed of the above two functional expressions is called the Exact Affine Stone Index Implicit Marshallian Demand System.

#### 2.2.3. Censoring Problem

If some households do not purchase all food items, the problem of censoring occurs. Because the data used in this analysis were collected using household surveys, it is common to observe zero values in the consumption of a particular food. In empirical studies, zero consumption has important econometric and economic implications, and statistical estimation procedures that do not account for these zero observations in the dependent variable have biased and inconsistent parameter estimates [37]. To address the problem of censoring in a reasonable way, this study applies the two-step consistent estimation developed by Shonkwiler and Yen (1999) in the second stage [38].

#### 2.2.4. Endogeneity

In the demand system model with expenditure share as the explained variable, the relationship between expenditure share, expenditure, and food price will cause endogeneity. This study uses the generalized method of moments (GMM) to estimate the two-stage models. To verify whether the endogeneity problem can be effectively dealt with after the above methods are applied, this study uses the Dubin–Wu–Hausman (DWH) test [3,30,39].

#### 2.2.5. Elasticity

Combining the condition expenditure elasticity and Marshall price elasticity in the two stages, building on the work of Carpentier and Guyomard (2001) [28], the unconditional expenditure elasticity (income elasticity) $e_{i}^{U}$ and unconditional Marshall price elasticity $e_{ij}^{U}$ of the two-stage EASI demand system model are calculated as:

(5) $$e_{i}^{U}=e_{i}\times e_{food}$$

(6) $$e_{ij}^{U}=e_{ij}+w_{j}\left[\frac{1}{e_{j}}+e_{food}^{p}\right]e_{i}e_{j}+e_{i}e_{food}w_{j}W_{f}\left(e_{j}-1\right)$$

where $e_{food}$ is the total food expenditure elasticity with respect to income in the first stage; $e_{i}$ is the conditional expenditure elasticity of food *i*; $e_{ij}$ is the conditional Marshall price elasticity of food *i* with respect to the price of food *j*; $e_{food}^{p}$ is the Marshallian self-price elasticity of total food consumption in the first stage; and $W_{f}$ is the expenditure share of the food group in total expenditure.

### 2.3. Dynamic Prediction Method

Compared with the traditional static simulation method, this method fully considers the heterogeneity and the dynamic mechanism across income groups and corrects the estimation bias caused by the use of average population elasticity and static simulation.

Prior to carrying out the simulation analysis, referring to Zheng and Henneberry (2010), Ren et al. (2018), and Li et al. (2021) [21,29,40], four basic assumptions are set. First, it is assumed that the consumer preferences of each income group will not change in the whole simulation process; that is, the income elasticity, price elasticity, income growth rate, and price change rate of a specific income group are fixed. Second, it is assumed that household structure also impacts food consumption. Since the household adult equivalence scale captures precise household structural characteristics, the household-level data in the sample are converted into standard human per capita food consumption through the household adult equivalence scale [41,42]. Third, the impact of factors other than income, price, and family structure on food consumption is not considered. Fourth, the total population is assumed to be constant, but the population of each income group is dynamic.

Based on the above assumptions, the per capita food consumption in period *t* + 1 can be expressed as:

(7) $$q_{i,t+1}=\underset{m}{\sum }\left\{\left[e_{m,\;i}{\left(\frac{\Delta Y_{t}}{Y_{t}}\right)}_{m}+\underset{j}{\sum }e_{m,\;ij}\left(\frac{\Delta P_{j,t}}{P_{j,t}}\right)+1\right]\cdot q_{m,i,t}EH_{m,t}\right\}/EH_{t}\;$$

where *t* = 0, …, 14; *i* and *j* represent thirteen food items; $q_{i,t+1}$ represents the per capita consumption of food *i* in period *t* + 1; $q_{m,i,t}$ is the per capita consumption of food *i* in period *t* by the residents of income Group *m*; *E* is the adult-equivalent household population; $H_{m,t}$ represents the number of households in income Group m in period *t*; $H_{t}$ is the total number of households in the sample in period *t*, $H_{t}=\overset{n}{\underset{m=1}{\sum }}H_{m,t}$; *n* is the number of divided income groups; $e_{m,\;i}$ represents the income elasticity of food *i* for residents of income Group *m*; $e_{m,\;ij}$ represents the unconditional Marshall price elasticity for residents in income Group *m*; ${\left(\Delta Y_{t}/Y_{t}\right)}_{m}$ represents the income growth rate of residents in income Group *m* in period *t*; and $\Delta P_{j,t}/P_{j,t}$ is the rate of change in the price of food *j* in period *t*.

**Figure 1:**
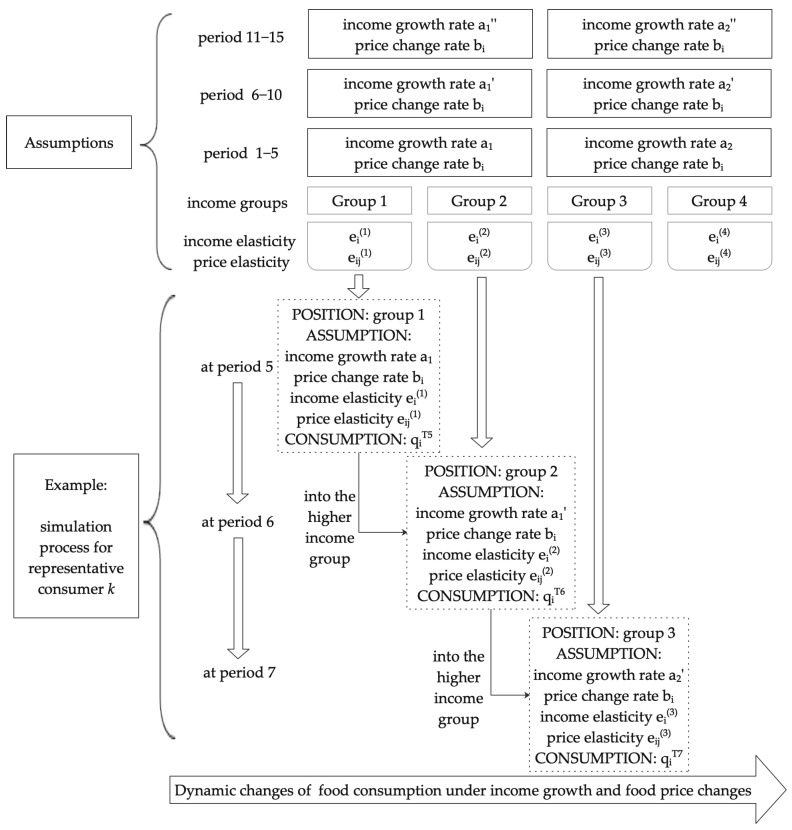
Dynamic simulation process for a representative consumer *k.* Notes: The ′ and ″ in this figure are just to indicate that the income growth rate is different in period 1–5, period 6–10 and period 11–15.

Specifically, the threshold value between the income groups is determined when dividing the income groups. If the consumer crosses the threshold between adjacent income groups after the current period’s income increase, the consumer will enter the adjacent higher income group during the following period’s simulation. Meanwhile, consumers will use the demand elasticity, income growth rate, and price change rate of their current income group in the simulation. Figure 1 displays an overview of the dynamics on food consumption of representative consumer *k*.

Taking the time of sample data as the base period, the changes in food consumption in simulation period 15 are extrapolated given the demand elasticity, income growth rate, and food price change rate. Among them, the demand elasticity of different income groups comes from the estimation results of the EASI model. Moreover, the income growth rate of different income groups is calculated based on data from 2009 to 2018 sourced from the National Bureau of Statistics. In simulation periods 1–5, the income growth rates of the low-income group, lower-middle-income group, middle-income group, upper-middle-income group, and high-income group are 6.66%, 6.94%, 7.28%, 7.44%, and 6.91%, respectively. Furthermore, the extrapolated income growth rate for the 6–10 period and 11–15 period will be reduced and raised by one percentage point, respectively. Additionally, based on the uncertainty and complexity of food price change, this study sets the scenario assumption of the price change rate based on previous research. The price change rates of rice, wheat, oils, pork, beef, mutton, poultry, eggs, dairy, seafood, vegetables, fruits, and other grains are 1.521, 1.015, 1.871, 0.283, 0.516, 0.216, 0.469, 1.099, 0.955, 0.985, −0.064, −0.064, and 1.268, respectively.

### 2.4. Data and Variables

#### 2.4.1. Data Collection

The data used in this study come from the urban household survey conducted by the National Bureau of Statistics of China. The NBSC household survey data reflect the regional differences through 22,210 urban households from Hebei Province (region: north), Jilin Province (northeast), Henan Province (central), Guangdong Province (south), Sichuan Province (southwest), and Xinjiang Uygur Autonomous Region (west). The advantage of this extensive dataset is that the chosen households in the survey recorded their food consumption and expenditure characteristics using a diary for over a year, which accurately captured the consumption behavior of Chinese households. The data are widely used in Chinese food consumption and related empirical studies [2,43,44,45]. Therefore, the dataset used in this study is of high quality and can more realistically reflect the dietary features across different income strata in China.

The standard for dividing the income groups in this study is to sort all sample families from low to high according to the level of household disposable income and divide them into five equal parts on average. Specifically, ranking all households in descending order of income, the low-income group refers to households in the lowest 20% income bracket, the high-income group refers to those in the highest 20%, and the lower-middle-income group, middle-income group, and upper-middle-income group refer to the remaining portion of the sample [5,46]. The division standard of 100 equal income groups is the same.

#### 2.4.2. Major Variables and Statistical Analysis

The food system of this study includes thirteen food items, namely, rice, wheat, oils, pork, beef, mutton, poultry, eggs, dairy, seafood, vegetables, fruits, and other grains. In the first stage, the budget share of food expenditure in the total expenditure is the dependent variable, while the shares of each food expenditure in total food expenditure are the dependent variable of the EASI model in the second stage. The proportion of various food expenditures in food expenditures ranges from 1.53% to 20.26% (Table 1).

Among the explanatory variables, income or expenditure is an essential determinant of food consumption. The average annual food expenditure of the sample households is RMB 6351.73, accounting for 20.55% of the total household consumption expenditure. The literature suggests that the Engel curve forms of various foods are not linear or even quadratic, and there may be higher-order Engel curve forms in practice [33]. Therefore, the Engel curve is set as a cubic type (fourth-order rank demand system) in the EASI model and is tested by the significance of the model coefficient $\alpha_{r}$.

To examine the differences in the food consumption status of residents in different income groups, this study uses a one-way analysis of variance (ANOVA) to carry out statistical inference. That is, through the F statistic and its significance level, it can be judged whether there is a difference in the overall mean represented by the mean food consumption of residents in different income groups (Table A1). There are significant differences in the consumption of various foods among different income groups at the 1% statistical significance level. In general, the consumption of most foods increases with income levels. This illustrates the importance of considering income group differences when estimating demand elasticity and simulation analysis.

In addition to other control variables except for income, this study also considers food prices and nine demographic variables and controls for time and region effects through year and province dummy variables (Table 1). First, since the dataset does not contain food price variables, the unit value obtained by dividing the expenditure by the consumption quantity is used [27,30,47]. Second, this study added three variables to reflect the impact of household structure on food consumption, including family size, the dummy of whether seniors are aged 65 and above, and the dummy of whether they have children aged 14 and below. Third, this study also added the education, age, and nationality of household food consumption decision-makers. Finally, this study incorporates household registration, characteristics of food consumption when away from home (FAFH), and the level of urbanization [48].

## 3. Results

### 3.1. Model Estimation Results

In the first stage, three Engel models were estimated by the least squares method (OLS) and the generalized method of moments (GMM) (Table A2). In all three models, the coefficients of the log of expenditures were significant at the 1% statistical significance level. However, the coefficients of the log of expenditures for Model 1 and Model 2 are negative because neither model considers the nonlinear effect of expenditures, which is not as expected. In contrast, Model 3 considers the nonlinear effects of expenditure while controlling for food prices and other goods prices. Although the coefficient of the log of expenditure in the OLS estimation result is still negative, after using the GMM method to deal with the endogeneity problem, the sign of the coefficient becomes positive. Additionally, the coefficient of the square term of the log of expenditure is also significant at the 1% statistical significance level, as expected. Furthermore, the food price index variables of Model 2 and Model 3 are both significant at the 1% level, but the sign of the coefficient is positive, consistent with the results of Ren et al. (2018) [40]. Considering that Model 3 uses the GMM estimation method to deal with the endogeneity effectively and includes the expenditure square term and the price index, this study uses the estimation results of Model 3 to calculate the elasticity.

In the second stage, the GMM method is used to estimate the EASI demand system model dealing with the problem of censoring and endogeneity. The estimated parameters are very complex, so this study only explains the statistical significance level of critical parameters, including expenditure and prices (Table A3). The estimation results show that 36 parameters of 48 expenditure items (α_i0_*, α_i_*_1_*, α_i_*_2_, and *α_i_*_3_, *i* = 1, …, 12) are significant at the 1% statistical significance level. Among them, 9 of the 12 parameters of the cubic term of expenditure (*α_i_*_3_, *i* = 1, …, 12) were significant at the 1% statistical significance level. These results prove that the Engel curves of most foods satisfy the cubic characteristics, and the rank of the demand system is higher than the third order. It can be seen that it is correct to select the EASI requirement system model in the second stage. Meanwhile, 9 parameters of the 12 own-price items (*A*_0*,ii*_, *i* = 1, …, 12) are significant at the 1% statistical significance level and 1 at the 5% statistical significance level. Of the other 66 cross-price parameters (*A*_0*,ij*_, *i* = 1, …, 12, *j* = 1, …, 12, *i* ≠ *j*), 37 parameters were statistically significant, at least at 1%.

The Dubin–Wu–Hausman test results showed that the values of the DWH statistic in the two stages were 35,976.9 and 4443.2, respectively, rejecting the null hypothesis at the 1% statistical significance level and proving that the endogeneity problem was effectively dealt with. Furthermore, the values of order one to order three of the Hessian matrix are 0.0654, 0.0018, and 0.0001, respectively. The values of the remaining k-order main subformulas are all 0, and the results are all non-negative values. Therefore, the EASI demand system model established in this study satisfies the demand property of negativity.

In summary, the parameters estimated by the two-stage EASI demand system model in this study are robust to a certain extent. Next, the income and price elasticity of food demand are estimated based on the estimated parameters.

### 3.2. Elasticity Estimation Results

For the whole sample, the results of the food demand elasticity estimated by the two-stage EASI model show that the income elasticity of various types of food for urban residents ranges from 0.392 to 0.743 (*p* < 0.01), and the unconditional Marshall own-price elasticity ranges from −1.558 to −0.664 (*p* < 0.01) (Table 2). The three food items with the most flexible income elasticity are beef, mutton, and seafood. The three food items with the most flexible own-price elasticity are wheat, rice, and poultry. The result indicates that these food items are most sensitive to income growth and price changes. Additionally, the conditional Marshall (uncompensated) price elasticity and conditional expenditure elasticity are provided in Table S1. The conditional Hicksian (compensated) price elasticity is provided in Table S2.

**Figure 2:**
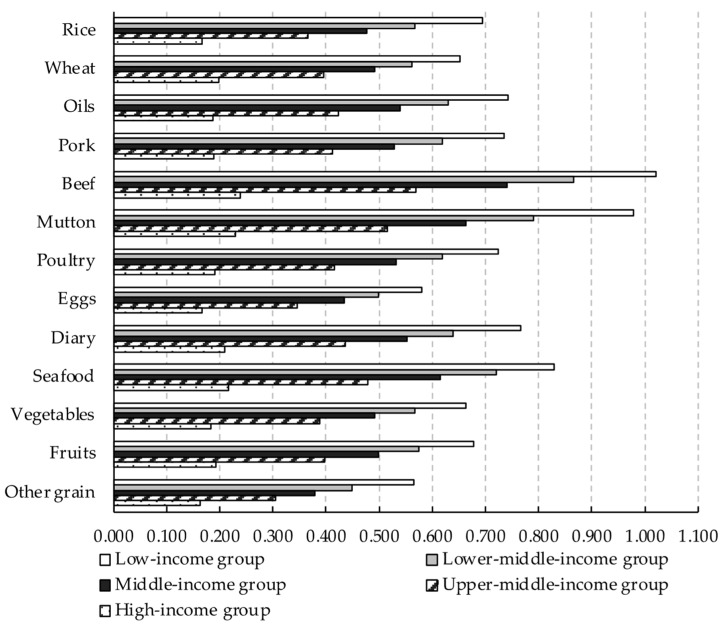
Income elasticity for different income groups estimated by the two-stage EASI demand system model.

**Figure 3:**
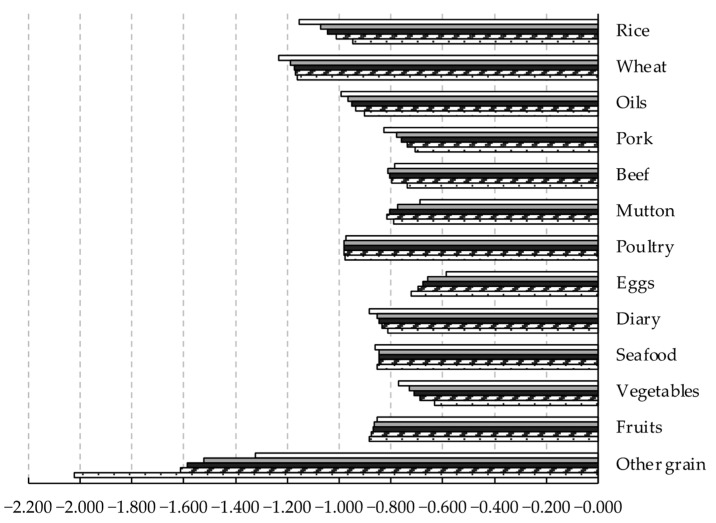
Own-price elasticity for different income groups estimated by the two-stage EASI demand system model.

**Figure A1:**
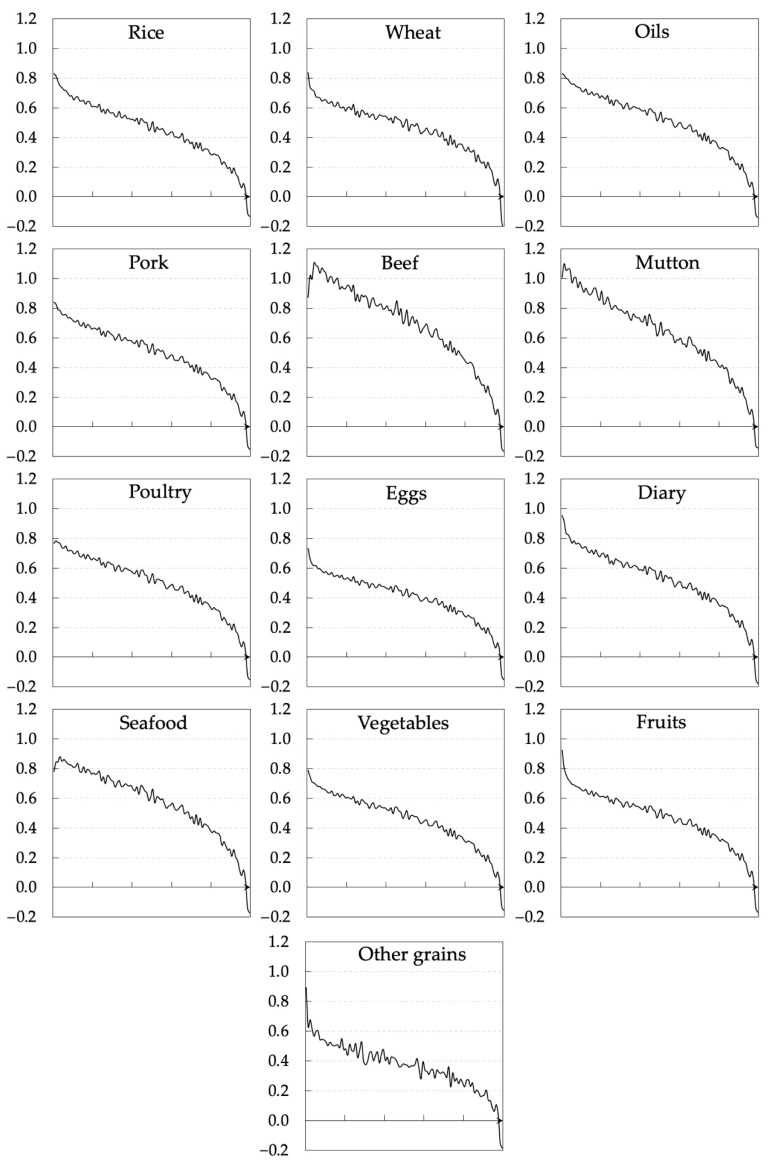
Income elasticity for 100 income groups. Notes: The horizontal axis of each subgraph represents an increase in income from left to right.

**Figure A2:**
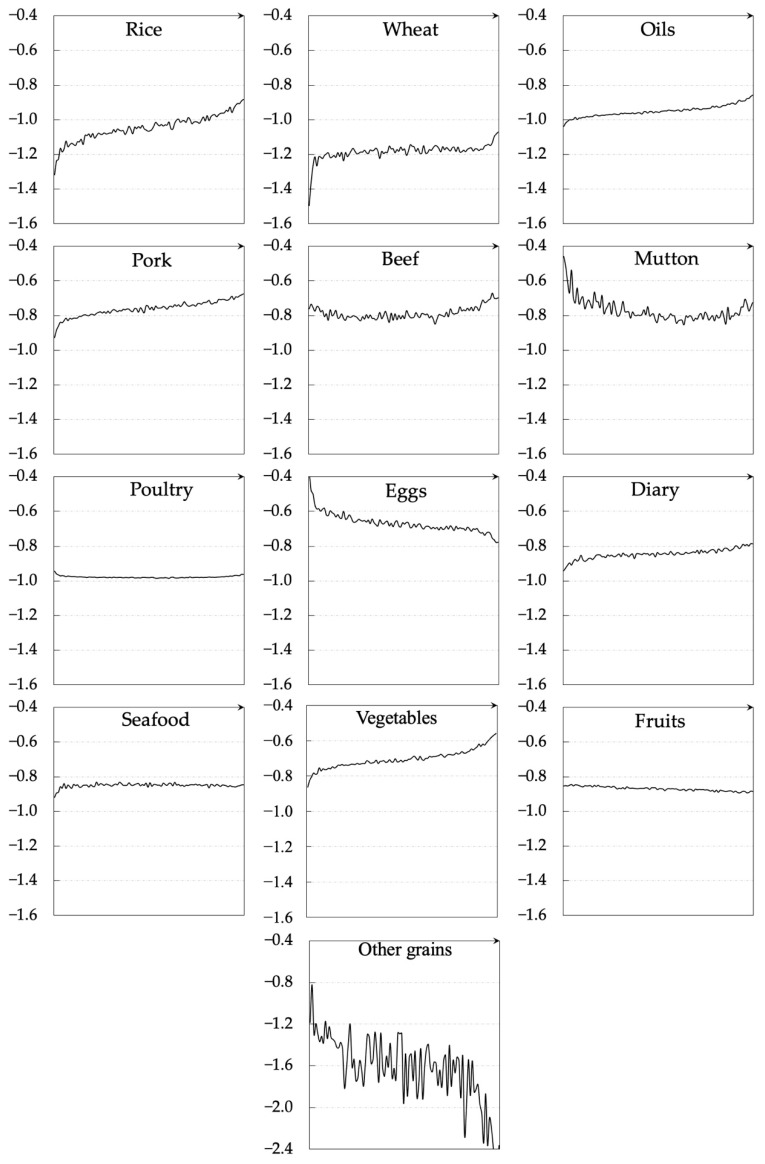
Own-price elasticity for 100 income groups. Notes: The horizontal axis of each subgraph represents an increase in income from left to right.

This study further calculates the food demand elasticity for different income groups. The most significant finding is that, with the increase in income, the income elasticity displays a gradually decreasing trend (Figure 2), but the own-price elasticity shows a differentiated trend (Figure 3). Among the income elasticity of the five income groups, the income elasticity of the low-income group is 0.565 to 1.020, while the income elasticity of the high-income group is narrowed to 0.162 to 0.239. The empirical results are consistent with the literature, which proves Engel’s law [20,45,49]. It should be noted that the distribution pattern of the income elasticity of various foods remained unchanged. Among the income elasticity of the low-income group, the income elasticity of beef is the most elastic, and the income elasticity of other grains is the least elastic. Meanwhile, the change characteristics of income and own-price elasticity with income growth of the 100 income groups are consistent with the five income groups (Figure A1 and Figure A2). The unconditional Marshall (uncompensated) price elasticity and the income elasticity of five income groups are provided in Tables S3–S7.

### 3.3. Simulation Results

#### 3.3.1. Food Consumption Perspectives

**Figure 4:**
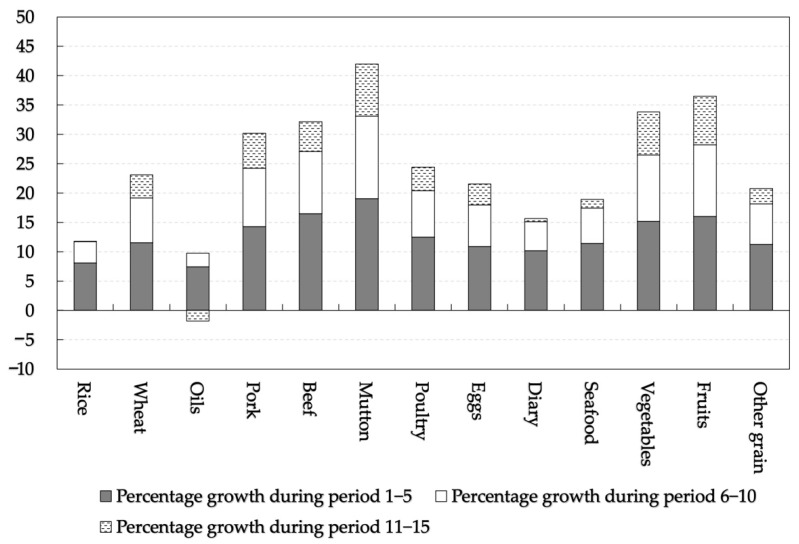
Percentage growth in food consumption after 15 periods. Notes: The change in food consumption is the percentage change from the sample base period.

Income growth and price changes will continue to increase the per capita food consumption of Chinese urban residents (Figure 4). The consumption of all food items in the 10th period will increase by 9.8% to 33.1% from the base period. Meanwhile, food consumption in the 15th period will increase by 7.9% to 42.0% over the base period. Among these periods, all foods show steady growth except rice, oil, and dairy, which follow a trend of growth followed by decline. The consumption of meat and fruits and vegetables grows faster than that of grains and other animal foods. In the 15th period, the increase in per capita food consumption in descending order is mutton, fruits, vegetables, beef, pork, poultry, wheat, eggs, other grains, seafood, dairy, rice, and oil. Specifically, the consumption of rice and oil increases first and then decreases, while the consumption of wheat and other grains continues to expand. In meat products, the consumption of pork, beef, mutton, and poultry shows a rapid growth trend, and the growth rate is ranked from high to low as mutton, beef, pork, and poultry. Meanwhile, the consumption of eggs, dairy, and seafood increases steadily. Among them, diary shows an increasing trend before decreasing. Additionally, the consumption of vegetables and fruits has risen rapidly, surpassing some animal products, and the growth rate of fruits is faster than that of vegetables.

#### 3.3.2. Impact of Income Heterogeneity on Food Prediction

**Figure A3:**
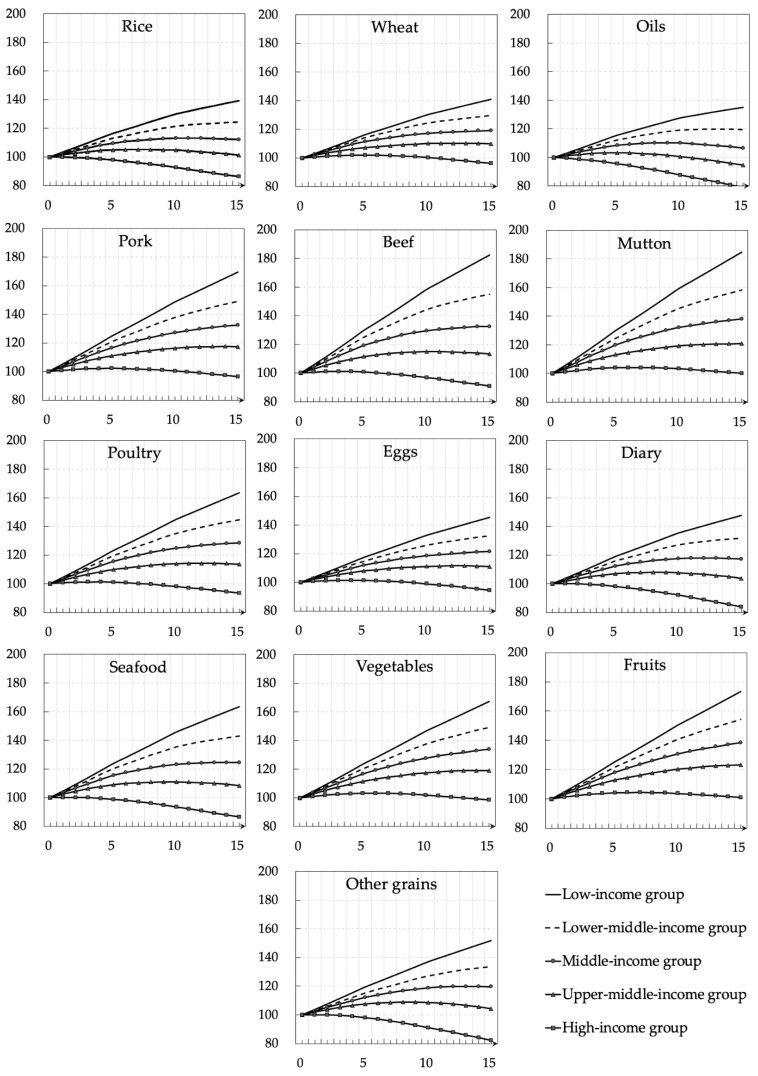
Simulation results of changes in food consumption for different income groups. Notes: The horizontal axis of each subgraph represents the predicting period from period 0 to period 15.

There are differences in the growth patterns of food consumption across income groups. The simulation results of the low-income, lower-middle-income, middle-income, upper-middle-income, and high-income groups are shown in Figure A3. On the one hand, the food consumption of the low-income, lower-middle-income, and middle-income groups will follow an increasing trend with income growth and price changes. Among them, the per capita consumption of the thirteen foods for residents in the low-income group will increase by 34.9% to 84.7% during the extrapolated 15-year period. The per capita consumption of the thirteen foods in the lower-middle-income group and the middle-income group will increase in the range of 19.6% to 58.3% and 6.5% to 38.4%, respectively, during the extrapolated 15-year period. On the other hand, the food consumption in the upper-middle-income group and the high-income group will show a pattern of either growth or decrease. Among them, in the simulation results of the upper-middle-income group, except for oils, which decrease by 5.3% in the 15th period compared with the base period, the per capita consumption of the other 12 foods will increase by 1.2% to 20.8% in the extrapolated 15th period. In the simulation results for the high-income group, mutton and fruit are the only two food items that show positive growth, with a growing range of 0.2% to 0.9%. The per capita consumption of the other ten food items will be lower than the base period levels in period 15, with a declining range of 1.5% to 20.7%.

Furthermore, this study analyzes the impact of the number of income groups and the dynamic procedure on the simulation results (Table 3). The Baseline Scenario, Scenario 1, and Scenario 2 have 100, 5, and 1 income groups, respectively. In other words, the Baseline scenario and Scenario 1 examine the impact of income heterogeneity on food consumption forecasting, while Scenario 2 continues to use the overall sample parameters for forecasting. Scenario 3 shows the simulation results of the traditional static procedure, which is used to compare and analyze the impact of the simulation method on the prediction results. The simulation results for the different scenarios show that the results are overestimated if the differences in consumer behavior between income groups are not considered. Additionally, the more detailed the division into income groups, the smaller the overestimation bias. The average percentage growth in per capita consumption of the 13 food items in the Baseline scenario, Scenario 1, and Scenario 2 are 24.5%, 28.1%, and 48.2% in period 15, respectively. However, if a dynamic procedure across income groups is not used in the simulation methodology, the prediction results will also be overestimated.

### 3.4. Robustness Check

To ensure the robustness of the simulation results, this study conducted a sensitivity analysis for the critical parameter for simulated food consumption changes based on the Baseline scenario (Table 4). When the value of all income and price elasticities decreases by 20% in sensitive Scenario 1, the mean value of the percentage growth in food consumption in the 15th period decreases by 5.7 percentage points. Meanwhile, when the value of all income and price elasticities increases by 20% in sensitive Scenario 2, the mean value of the percentage growth in food consumption in period 15 increases by 6.1 percentage points. The sensitivity analysis demonstrates the robustness of the simulation results.

## 4. Discussion

Recently, investigators have examined the impact of income on food consumption and estimated income elasticity based on the EASI model [6,35]. However, there is a general paucity of empirical research that seeks to identify the predictions of food consumption systematically considering the dynamic impacts of income difference. Therefore, this study makes a major contribution to research on food consumption in urban China by demonstrating the dynamic impact of income heterogeneity based on an extensive dataset and the EASI demand system. China’s food consumption will continue to grow in the future. Nevertheless, different categories of food will have different growth patterns. Excluding differences in consumption behavior between income groups would significantly overestimate the predicted results for food consumption. Inaccurate demand forecasts could mislead agricultural production. Meanwhile, the inequality of income distribution in China is changing dramatically. China’s Gini coefficient, a measure of the unequal distribution of population income, was 0.490 in 2009 and 0.468 in 2020, reflecting significant income disparity in China. Therefore, in addition to the significant changes in the income distribution of Chinese residents, the effects of income heterogeneity persist over time and profoundly affect the dietary transition of Chinese residents. The impact of income heterogeneity on food consumption cannot be ignored.

Considering the predicted results of grains and oil, it can be seen that the consumption of rice and wheat does not show a continuous downward trend. However, rice consumption declined after a steady increase, and wheat consumption continued to grow. The growth in demand for wheat may originate from the westernization and diversification of the dietary patterns of Chinese residents in different regions [50,51]. Furthermore, the challenges posed by the growth in grain consumption demand to China’s food security requires attention. Meanwhile, the trend in the consumption of oils and other grains may be related to the improved health perceptions of the population. According to the latest version of the *Chinese Dietary Guidelines (2022)*, reducing fat intake and eating more mixed grains are beneficial for health management [52].

The predicted results for animal source foods show that eggs, as the most widely consumed source of daily protein intake by Chinese residents, will show a rigid growth trend in consumption demand. Additionally, beef, mutton, dairy, and seafood are high-quality products and essential sources of high-quality protein in the diet [2]. The growth in demand for these four food items illustrates the growing diversified dietary needs of the Chinese population, which indicates that the Chinese dietary transition will continue to shift from the traditional fiber-dominated food system to a Western-style meat-dominated diet [1,4,10]. Furthermore, the increase in greenhouse gas emissions brought by increasing meat consumption and its negative impact on the global environment cannot be ignored.

Furthermore, the predicted expansion of consumption for vegetables and fruits proves that the Chinese population is gradually pursuing a healthier diet. This rising consumption of fruits and vegetables is because vegetables and fruits are rich in vitamins, minerals, and dietary fiber and are low in energy, which has important effects such as reducing the risk of chronic diseases such as cardiovascular diseases and cancer [52,53,54]. With the further improvement of Chinese residents’ income level and health concepts, as well as the decrease in fruit and vegetable prices due to the scale effect and technological progress, healthy and nutritious food with low calories, low fat, and high fiber will become not only a fashion label but also a general lifestyle. Fruits and vegetables will play an increasingly important role in the future dietary structure of Chinese residents.

Several important policy implications are derived from the study. First, since income heterogeneity significantly impacts the prediction results of food consumption, the differences in consumer behavior of different income groups should be fully incorporated into the early warning and prediction research of food consumption. Second, since the food consumption growth potential of low-income groups is significantly higher than that of middle- and high-income groups, joint measures should be taken from the production and import sides to ensure the primary food supply of low-income groups and improve the diet quality of middle- and high-income groups. Third, considering future trends, the structure of food consumption in China will continue to be transformed and upgraded, which will bring about unbalanced nutritional intake and negative environmental impacts. In this context, exploring the transformation path of the food system has become an essential topic in the development of human society [9]. It is an important policy tool to guide residents to transition to new sustainable dietary patterns by strengthening publicity and other options [16,55].

## 5. Conclusions

In summary, based on nationwide data and the two-stage EASI demand system model, this paper provides insights into the impacts of income heterogeneity on the demand elasticity and prediction of food consumption in urban China. We found that income elasticity shows a decreasing trend with income growth. Furthermore, the consumption of major food items will continue to expand in the future. Nevertheless, there are different growth patterns across income groups. The most obvious finding to emerge from the analysis is that the income elasticity of food consumption displays a gradually decreasing trend with the increase in income, and food consumption of lower income groups increased more than higher income groups during the extrapolated 15-year period. Therefore, the empirical results as well as the simulation results both indicate that food consumption growth potential of low-income groups is significantly higher than that of middle- and high-income groups. However, the prediction results will be overestimated if the differences in consumer behavior across income groups and the dynamic simulation procedure are not taken into account. These findings may further indicate that the consumer characteristics of different income groups need to be included in food consumption forecasts. Moreover, the government should formulate food policies for different income groups to promote a sustainable transformation of the food system. Specifically, on the one hand, it is necessary to meet the food intake of low-income groups by improving the total food supply capacity and emergency food distribution. Food subsidies can be appropriately given to vulnerable groups on the verge of returning to poverty. On the other hand, the transition to healthy diets for middle-income groups should be promoted through dietary guidelines and advertising. In addition, it is recommended that the government should guide urban high-income consumers to further consider the low-carbon attributes of food while ensuring a healthy diet. For example, the food carbon footprint can be reduced by reducing red meat consumption and increasing local vegetable intake.

## Figures and Tables

**Table 1:** Summary statistics for major variables.

Variables	Mean	S.D.	Variables	Mean	S.D.
Budget share (%)			Social economic variables		
Rice in food expenditure	6.61	3.17	Rice price (kg/RMB)	3.34	0.37
Wheat in food expenditure	2.29	3.07	Wheat price (kg/RMB)	0.67	0.50
Oils in food expenditure	7.07	3.89	Oils price (kg/RMB)	5.34	2.49
Pork in food expenditure	20.26	7.83	Pork price (kg/RMB)	12.46	1.59
Beef in food expenditure	3.01	2.68	Beef price (kg/RMB)	16.60	3.32
Mutton in food expenditure	2.02	3.50	Mutton price (kg/RMB)	19.15	4.12
Poultry in food expenditure	10.22	5.29	Poultry price (kg/RMB)	9.27	9.09
Eggs in food expenditure	4.56	2.60	Eggs price (kg/RMB)	5.92	0.79
Diary in food expenditure	7.24	4.87	Diary price (kg/RMB)	3.06	1.49
Seafood in food expenditure	6.86	4.91	Seafood price (kg/RMB)	5.53	2.74
Vegetables in food expenditure	18.36	5.25	Vegetables price (kg/RMB)	2.55	0.47
Fruits in food expenditure	9.99	4.69	Fruits price (kg/RMB)	1.90	0.79
Other grains in food expenditure	1.53	1.12	Other grains price (kg/RMB)	2.28	0.78
Food in total expenditure	20.55	10.59	Food expenditure (RMB)	6351.73	3504.36
Demographic variables			Region and time dummy variables		
Family size	2.93	0.92	Guangdong (Yes = 1; No = 0)	0.28	0.45
Seniors aged 65 and above (Yes = 1; No = 0)	0.35	0.48	Sichuan(Yes = 1; No = 0)	0.23	0.42
Children aged 14 and below (Yes = 1; No = 0)	0.21	0.41	Jilin (Yes = 1; No = 0)	0.04	0.21
Proportion of FAFH (%)	5.11	5.21	Hebei (Yes = 1; No = 0)	0.17	0.38
Local urban household registration (Yes = 1; No = 0)	0.94	0.24	Henan (Yes = 1; No = 0)	0.22	0.41
High school (Yes = 1; No = 0)	0.34	0.47	Xinjiang (Reference)	0.06	0.25
Age (years old)	47.72	12.27	Year 2007 (Reference)	0.20	0.40
Han nationality (Yes = 1; No = 0)	0.98	0.15	Year 2008 (Yes = 1; No = 0)	0.38	0.49
Town size (small = 1; middle = 2; big = 3)	1.87	0.53	Year 2009 (Yes = 1; No = 0)	0.42	0.49

Notes: Education, age, and ethnicity refer to the meal planner’s feature.

**Table 2:** Unconditional Marshall (uncompensated) price elasticity and income elasticity.

	Rice	Wheat	Oils	Pork	Beef	Mutton	Poultry	Eggs	Diary	Seafood	Vegetables	Fruits	Other Grains
Unconditional price elasticity													
Rice	−1.049 ***	0.012 ***	−0.002 ***	0.017 ***	−0.003 ***	0.021 ***	0.020 ***	−0.075 ***	−0.004 ***	−0.036 ***	−0.081 ***	0.021 ***	0.428 ***
Wheat	0.027 ***	−1.189 ***	−0.024 ***	0.044 ***	0.038 ***	0.036 ***	0.059 ***	−0.078 ***	−0.010 ***	0.006 ***	0.148 ***	0.010 ***	0.190 ***
Oils	−0.019 ***	−0.004 ***	−0.955 ***	0.043 ***	−0.008 ***	0.005 ***	0.016 ***	−0.010 ***	0.020 ***	−0.005 ***	0.009 ***	0.025 ***	0.060 ***
Pork	0.041 ***	0.012 ***	0.014 ***	−0.764 ***	−0.002 ***	−0.020 ***	0.016 ***	−0.033 ***	−0.005 ***	−0.014 ***	−0.051 ***	−0.019 ***	0.019 ***
Beef	0.225 ***	0.010 ***	0.032 ***	0.032 ***	−0.792 ***	−0.017 ***	0.063 ***	0.072 ***	−0.025 ***	0.058 ***	0.090 ***	0.037 ***	−0.911 ***
Mutton	−0.005 ***	0.019 ***	0.047 ***	−0.154 ***	−0.003 **	−0.775 ***	0.044 ***	0.145 ***	−0.019 ***	0.052 ***	0.041 ***	0.059 ***	−0.491 ***
Poultry	0.026 ***	0.015 ***	0.011 ***	0.032 ***	0.013 ***	0.007 ***	−0.982 ***	0.007 ***	0.002 ***	0.011 ***	0.038 ***	0.019 ***	−0.010 ***
Eggs	−0.108 ***	0.006 ***	−0.021 ***	−0.141 ***	−0.044 ***	−0.009 ***	0.018 ***	−0.664 ***	−0.008 ***	−0.024 ***	−0.112 ***	0.010 ***	0.442 ***
Diary	−0.027 ***	−0.005 ***	0.011 ***	0.092 ***	−0.018 ***	−0.017 ***	0.005 ***	0.193 ***	−0.845 ***	0.032 ***	0.113 ***	0.074 ***	−0.455 ***
Seafood	−0.023 ***	0.010 ***	−0.007 ***	−0.045 ***	−0.008 ***	0.001 **	0.015 ***	−0.018 ***	−0.002 ***	−0.854 ***	−0.049 ***	0.041 ***	0.007 ***
Vegetables	−0.035 ***	0.005 ***	0.004 ***	−0.054 ***	−0.025 ***	−0.005 ***	0.022 ***	−0.028 ***	−0.004 ***	−0.017 ***	−0.707 ***	−0.009 ***	0.106 ***
Fruits	0.034 ***	0.004 ***	0.018 ***	−0.037 ***	−0.024 ***	0.004 ***	0.020 ***	0.004 ***	0.023 ***	0.030 ***	−0.016 ***	−0.870 ***	0.048 ***
Other grains	1.854 ***	0.285 ***	0.278 ***	0.257 ***	−1.792 ***	−0.649 ***	−0.066 ***	1.319 ***	−2.154 ***	0.035 ***	1.279 ***	0.318 ***	−1.558 ***
Income elasticity	0.483 ***	0.491 ***	0.545 ***	0.533 ***	0.743 ***	0.687 ***	0.536 ***	0.433 ***	0.560 ***	0.616 ***	0.493 ***	0.503 ***	0.392 ***

Notes: ** *p* < 0.05, *** *p* < 0.01.

**Table 3:** Percentage growth in food consumption after period 15 under different scenarios.

Items	Baseline Scenario	Scenario 1	Scenario 2	Scenario 3
Income groups	100 groups	5 groups	1 group	100 groups
Income heterogeneity	Yes	Yes	No	Yes
Dynamic procedure	Yes	Yes	Yes	No
Rice	11.8	14.8	30.1	26.5
Wheat	23.1	23.9	33.9	35.1
Oils	7.9	10.3	26.2	23.5
Pork	30.2	34.8	57.4	50.8
Beef	32.1	36.3	66.9	59.1
Mutton	42.0	44.3	70.7	67.6
Poultry	24.4	29.5	52.8	44.3
Eggs	21.6	24.2	38.0	35.7
Diary	15.6	19.5	39.0	33.9
Seafood	18.9	24.7	51.5	41.2
Vegetables	33.8	37.4	56.8	53.3
Fruits	36.5	40.9	62.5	57.4
Other grains	20.7	24.8	41.1	39.8
Average	24.5	28.1	48.2	43.7

Notes: The change in food consumption is the percentage change from the sample base period.

**Table 4:** Sensitivity analysis of demand elasticity.

Items	Baseline Scenario with Original Elasticity	Sensitive Scenario 1 with All Elasticity Reduced by 20%		Sensitive Scenario 2 with All Elasticity Increased by 20%	
	Percentage Growth (%)	Percentage Growth (%)	Deviation	Percentage Growth (%)	Deviation
Rice	11.8	9.1	−2.7	14.6	2.8
Wheat	23.1	18.0	−5.1	28.4	5.3
Oils	7.9	6.0	−1.9	10.0	2.1
Pork	30.2	23.2	−7.0	37.7	7.5
Beef	32.1	24.5	−7.6	40.5	8.4
Mutton	42.0	32.1	−9.9	52.8	10.8
Poultry	24.4	18.8	−5.6	30.5	6.1
Eggs	21.6	16.7	−4.9	26.6	5.0
Diary	15.6	12.0	−3.6	19.5	3.9
Seafood	18.9	14.5	−4.4	23.8	4.9
Vegetables	33.8	26.0	−7.8	42.2	8.4
Fruits	36.5	28.0	−8.5	45.6	9.1
Other grains	20.7	15.9	−4.8	25.9	5.2
Average	24.5	18.8	−5.7	30.6	6.1

Notes: The change in food consumption is the percentage change from the sample base period.

## Data Availability

The data presented in this study are available upon request from the corresponding author.

## Supplementary Materials

The following supporting information can be downloaded: https://www.mdpi.com/article/10.3390/foods11172597/s1, Table S1: Conditional Marshall price elasticity and conditional expenditure elasticity (full sample); Table S2: Conditional Hicksian price elasticity (full sample); Table S3: Unconditional Marshall price elasticity and income elasticity (low-income group); Table S4: Unconditional Marshall price elasticity and income elasticity (lower-middle-income group); Table S5: Unconditional Marshall price elasticity and income elasticity (middle-income group); Table S6: Unconditional Marshall price elasticity and income elasticity (upper-middle-income group); Table S7: Unconditional Marshall price elasticity and income elasticity (high-income group).

## Appendix A

**Table A1:** Average food consumption by income group (kg/year).

Food Items	Low-Income Group	Lower-Middle-Income Group	Middle-Income Group	Upper-Middle-Income Group	High-Income Group	F-Statistic
Rice	91.80	109.60	116.77	122.11	143.86	242.40 ***
	(65.10)	(75.28)	(84.60)	(86.95)	(91.07)	
Wheat	41.99	46.68	46.88	41.21	25.71	103.45 ***
	(59.94)	(59.80)	(61.09)	(55.65)	(46.10)	
Oils	26.01	30.29	31.60	31.42	31.81	74.06 ***
	(16.72)	(17.80)	(19.41)	(19.49)	(20.34)	
Pork	44.70	55.92	61.95	66.05	83.90	552.00 ***
	(31.11)	(36.66)	(41.92)	(43.59)	(48.88)	
Beef	4.58	6.40	7.01	7.75	8.70	258.02 ***
	(5.10)	(6.26)	(6.46)	(6.96)	(7.14)	
Mutton	2.84	3.98	4.39	4.47	3.44	49.79 ***
	(5.80)	(6.75)	(7.20)	(6.93)	(5.43)	
Poultry	21.40	28.41	31.66	35.61	49.30	732.64 ***
	(17.22)	(22.01)	(25.07)	(27.86)	(32.72)	
Eggs	25.86	32.76	34.51	35.44	33.65	182.75 ***
	(17.25)	(19.09)	(19.25)	(19.88)	(18.45)	
Diary	45.11	61.86	68.75	75.02	78.87	302.40 ***
	(41.26)	(49.17)	(52.22)	(54.08)	(56.81)	
Seafood	18.03	24.90	29.67	34.46	48.57	773.34 ***
	(16.83)	(22.09)	(27.40)	(31.38)	(35.82)	
Vegetables	301.04	368.95	391.33	401.08	409.78	324.62 ***
	(149.93)	(152.54)	(161.81)	(168.25)	(175.54)	
Fruits	104.85	139.33	151.56	162.82	176.32	476.29 ***
	(72.65)	(81.59)	(84.70)	(87.43)	(88.48)	
Other grains	27.69	30.67	30.63	30.65	28.95	14.64 ***
	(24.15)	(24.30)	(23.92)	(23.79)	(21.57)	

Notes: Standard deviations in parentheses; *** *p* < 0.01.

**Table A2:** Estimated results of the first-stage Engel model.

Variable	Model 1		Model 2		Model 3	
	OLS	GMM	OLS	GMM	OLS	GMM
log expenditure	−0.104 ***	−0.096 ***	−0.104 ***	−0.094 ***	−0.109 ***	0.187 ***
	(0.001)	(0.001)	(0.001)	(0.001)	(0.017)	(0.030)
Square of log expenditure					0.0003	−0.014 ***
					(0.001)	(0.001)
Food price index			0.043 ***	0.043 ***	0.043 ***	0.041 ***
			(0.002)	(0.002)	(0.002)	(0.002)
Other good price index			0.008 ***	0.007 ***	0.008 ***	0.007 ***
			(0.001)	(0.002)	(0.001)	(0.002)
Family size	0.011 ***	0.010 ***	0.011 ***	0.010 ***	0.011 ***	0.010 ***
	(0.001)	(0.001)	(0.001)	(0.001)	(0.001)	(0.001)
Seniors aged 65 and above	0.003 **	0.003 ***	0.004 ***	0.004 ***	0.004 ***	0.005 ***
	(0.001)	(0.001)	(0.001)	(0.001)	(0.001)	(0.001)
Children aged 14 and below	0.014 ***	0.015 ***	0.013 ***	0.014 ***	0.013 ***	0.014 ***
	(0.001)	(0.001)	(0.001)	(0.001)	(0.001)	(0.001)
Proportion of FAFH	−0.003 ***	−0.003 ***	−0.003 ***	−0.003 ***	−0.003 ***	−0.003 ***
	(0.000)	(0.000)	(0.000)	(0.000)	(0.000)	(0.000)
Local urban household registration	0.007 ***	0.006 ***	0.005 **	0.004 **	0.005 **	0.004 *
	(0.002)	(0.002)	(0.002)	(0.002)	(0.002)	(0.002)
Education in high school	−0.014 ***	−0.017 ***	−0.014 ***	−0.017 ***	−0.014 ***	−0.016 ***
	(0.001)	(0.001)	(0.001)	(0.001)	(0.001)	(0.001)
Age of meal planner	0.001 ***	0.001 ***	0.001 ***	0.001 ***	0.001 ***	0.001 ***
	(0.000)	(0.000)	(0.000)	(0.000)	(0.000)	(0.000)
Han nationality of meal planner	0.002	0.004	0.003	0.004	0.003	0.003
	(0.003)	(0.003)	(0.003)	(0.003)	(0.003)	(0.003)
Town size	0.000	0.000	−0.001	0.000	−0.001	−0.001
	(0.001)	(0.001)	(0.001)	(0.001)	(0.001)	(0.001)
Guangdong	0.086 ***	0.084 ***	0.073 ***	0.071 ***	0.073 ***	0.075 ***
	(0.002)	(0.002)	(0.002)	(0.002)	(0.002)	(0.002)
Sichuan	0.045 ***	0.047 ***	0.038 ***	0.039 ***	0.038 ***	0.040 ***
	(0.002)	(0.002)	(0.002)	(0.002)	(0.002)	(0.002)
Jilin	0.003	0.004	0.002	0.002	0.002	0.004
	(0.003)	(0.003)	(0.003)	(0.003)	(0.003)	(0.003)
Hebei	−0.010 ***	−0.009 ***	−0.001	−0.002	−0.001	−0.003
	(0.002)	(0.002)	(0.002)	(0.002)	(0.002)	(0.002)
Henan	−0.028 ***	−0.027 ***	−0.027 ***	−0.026 ***	−0.027 ***	−0.027 ***
	(0.002)	(0.002)	(0.002)	(0.002)	(0.002)	(0.002)
Year 2008	0.015 ***	0.015 ***	0.002	0.004 **	0.002	0.005 **
	(0.001)	(0.001)	(0.002)	(0.002)	(0.002)	(0.002)
Year 2009	0.022 ***	0.021 ***	0.007 **	0.008 ***	0.007 **	0.009 ***
	(0.001)	(0.001)	(0.003)	(0.003)	(0.003)	(0.003)
Constant	1.171 ***	1.091 ***	1.082 ***	0.994 ***	1.110 ***	−0.437 ***
	(0.011)	(0.015)	(0.013)	(0.015)	(0.091)	(0.155)

Notes: Standard errors in parentheses; * *p* < 0.10, ** *p* < 0.05, *** *p* < 0.01.

**Table A3:** Estimated results of expenditure and price parameters for the second stage of the EASI demand system model.

	Coef.	S.E.		Coef.	S.E.		Coef.	S.E.		Coef.	S.E.		Coef.	S.E.		Coef.	S.E.
*α*_10_	−0.195	0.261	*α*_61_	−0.301 ***	0.058	*α*_112_	−0.082 ***	0.018	*A*_0*,*25_	0.021 ***	0.005	*A*_0*,*47_	−0.029 ***	0.007	*A*_0*,*77_	0.014 ***	0.005
*α*_11_	0.034	0.099	*α*_62_	0.047 ***	0.008	*α*_113_	0.004 ***	0.001	*A*_0*,*26_	0.001	0.005	*A*_0*,*48_	0.002	0.017	*A*_0*,*78_	−0.004	0.003
*α*_12_	0.005	0.013	*α*_63_	−0.002 ***	0.000	*α*_120_	−1.528 ***	0.255	*A*_0*,*27_	−0.001	0.003	*A*_0*,*49_	0.008	0.012	*A*_0*,*79_	−0.002	0.005
*α*_13_	−0.001	0.001	*α*_70_	1.073 ***	0.194	*α*_121_	0.739 ***	0.109	*A*_0*,*28_	0.000	0.004	*A*_0*,*410_	0.044 ***	0.012	*A*_0*,*710_	0.001	0.004
*α*_20_	−0.648	0.160	*α*_71_	−0.429 ***	0.084	*α*_122_	−0.109 ***	0.016	*A*_0*,*29_	0.007	0.005	*A*_0*,*411_	0.139 ***	0.028	*A*_0*,*711_	−0.017 ***	0.005
*α*_21_	0.244 ***	0.068	*α*_72_	0.062 ***	0.012	*α*_123_	0.005 ***	0.001	*A*_0*,*210_	−0.033 ***	0.005	*A*_0*,*412_	−0.031 * *	0.015	*A*_0*,*712_	−0.008 *	0.005
*α*_22_	−0.032 ***	0.010	*α*_73_	−0.003 ***	0.001	*A*_0*,*11_	−0.167 ***	0.034	*A*_0*,*211_	0.037 ***	0.007	*A*_0*,*55_	0.045 ***	0.011	*A*_0*,*88_	0.081 ***	0.018
*α*_23_	0.002 ***	0.000	*α*_80_	−0.635 ***	0.133	*A*_0*,*12_	−0.011 *	0.006	*A*_0*,*212_	0.017 ***	0.005	*A*_0*,*56_	−0.033 ***	0.009	*A*_0*,*89_	−0.011 **	0.004
*α*_30_	0.800 ***	0.232	*α*_81_	0.307 ***	0.055	*A*_0*,*13_	−0.015 *	0.008	*A*_0*,*33_	−0.039 ***	0.011	*A*_0*,*57_	0.019 ***	0.004	*A*_0*,*810_	−0.024 ***	0.005
*α*_31_	−0.341 ***	0.096	*α*_82_	−0.048 ***	0.008	*A*_0*,*14_	0.110 ***	0.026	*A*_0*,*34_	0.051 ***	0.014	*A*_0*,*58_	−0.006	0.009	*A*_0*,*811_	−0.032 **	0.013
*α*_32_	0.052 ***	0.014	*α*_83_	0.002 ***	0.000	*A*_0*,*15_	0.026 **	0.013	*A*_0*,*35_	−0.005	0.007	*A*_0*,*59_	0.003	0.006	*A*_0*,*812_	−0.008	0.006
*α*_33_	−0.003 ***	0.001	*α*_90_	−0.301	0.288	*A*_0*,*16_	−0.012	0.012	*A*_0*,*36_	0.004	0.008	*A*_0*,*510_	−0.018 ***	0.006	*A*_0*,*99_	−0.004	0.011
*α*_40_	0.367	0.304	*α*_91_	0.157	0.124	*A*_0*,*17_	0.019 ***	0.004	*A*_0*,*37_	0.011 ***	0.004	*A*_0*,*511_	0.000	0.013	*A*_0*,*910_	0.017 **	0.007
*α*_41_	−0.172	0.131	*α*_92_	−0.023	0.018	*A*_0*,*18_	−0.058 ***	0.018	*A*_0*,*38_	−0.021 ***	0.005	*A*_0*,*512_	−0.021 ***	0.007	*A*_0*,*911_	−0.043 ***	0.010
*α*_42_	0.024	0.019	*α*_93_	0.001	0.001	*A*_0*,*19_	0.012	0.007	*A*_0*,*39_	0.029 ***	0.007	*A*_0*,*66_	0.059 ***	0.011	*A*_0*,*912_	0.016 *	0.008
*α*_43_	−0.001	0.001	*α*_100_	1.091 ***	0.231	*A*_0*,*110_	0.023 ***	0.008	*A*_0*,*310_	−0.004	0.006	*A*_0*,*67_	0.014 ***	0.004	*A*_0*,*1010_	−0.025 ***	0.009
*α*_50_	1.267 ***	0.192	*α*_101_	−0.425 ***	0.102	*A*_0*,*111_	−0.018	0.020	*A*_0*,*311_	−0.031 ***	0.011	*A*_0*,*68_	0.001	0.007	*A*_0*,*1011_	−0.034 ***	0.010
*α*_51_	−0.559 ***	0.085	*α*_102_	0.058 ***	0.015	*A*_0*,*112_	0.043 ***	0.011	*A*_0*,*312_	0.017 **	0.008	*A*_0*,*69_	−0.007	0.007	*A*_0*,*1012_	0.018 **	0.008
*α*_52_	0.081 ***	0.012	*α*_103_	−0.003 ***	0.001	*A*_0*,*22_	−0.048 ***	0.004	*A*_0*,*44_	−0.148 ***	0.046	*A*_0*,*610_	−0.004	0.007	*A*_0*,*1111_	−0.067 **	0.030
*α*_53_	−0.004 ***	0.001	*α*_110_	−0.808 ***	0.293	*A*_0*,*23_	−0.012 **	0.005	*A*_0*,*45_	0.014	0.018	*A*_0*,*611_	0.006	0.013	*A*_0*,*1112_	−0.073 ***	0.012
*α*_60_	0.641 ***	0.142	*α*_111_	0.526 ***	0.125	*A*_0*,*24_	0.011	0.009	*A*_0*,*46_	0.013	0.018	*A*_0*,*612_	0.005	0.008	*A*_0*,*1212_	0.019	0.013

Notes: * *p* < 0.10, ** *p* < 0.05, *** *p* < 0.01. *α* is an element of vector $\alpha_{r}$ and *A* is an element of vector $A_{l}$ which are parameters of EASI demand system model.
