# Supplementary material for: How Does Income Heterogeneity Affect Future Perspectives on Food Consumption? Empirical Evidence from Urban China
Source: Foods. 2022 Aug 26;11(17):2597. doi: 10.3390/foods11172597 (PMC9455673; doi:10.3390/foods11172597)
Supplement: Supplementary file 1 [file foods-11-02597-s001.zip › foods-1846610-supplementary.pdf]

Table S1. Conditional Marshall price elasticity and conditional expenditure elasticity (full sample)

	Rice	Wheat	Oils	Pork	Beef	Mutton	Poultry	Eggs	Diary	Seafood	Vegetables	Fruits	Other grains
Conditional price elasticity													
Rice	**-1.065*****	0.007***	-0.014***	-0.019***	-0.001*	0.021***	0.002***	-0.089***	-0.014***	-0.041***	-0.122***	-0.001	0.423***
Wheat	0.011***	**-1.194*****	-0.036***	0.007***	0.041***	0.037***	0.041***	-0.091***	-0.021***	0.000	0.106***	-0.012***	0.185***
Oils	-0.037***	-0.010***	**-0.968*****	0.002***	-0.005***	0.005***	-0.004***	-0.025***	0.008***	-0.011***	-0.037***	0.001***	0.054***
Pork	0.023***	0.006***	0.001***	**-0.804*****	0.001***	-0.020***	-0.003***	-0.047***	-0.017***	-0.020***	-0.096***	-0.042***	0.013***
Beef	0.201***	0.002***	0.014***	-0.023***	**-0.788*****	-0.017***	0.036***	0.051***	-0.041***	0.050***	0.026***	0.005***	-0.919***
Mutton	-0.027***	0.012***	0.031***	-0.205***	0.000	**-0.775*****	0.019***	0.126***	-0.034***	0.045***	-0.018***	0.029***	-0.498***
Poultry	0.009***	0.010***	-0.002***	-0.008***	0.016***	0.007***	**-1.002*****	-0.008***	-0.010***	0.005***	-0.008***	-0.005***	-0.016***
Eggs	-0.122***	0.001***	-0.031***	-0.173***	-0.042***	-0.009***	0.002***	**-0.676*****	-0.018***	-0.029***	-0.149***	-0.009***	0.437***
Diary	-0.045***	-0.011***	-0.002***	0.050***	-0.015***	-0.017***	-0.015***	0.177***	**-0.857*****	0.025***	0.065***	0.049***	-0.461***
Seafood	-0.043***	0.003***	-0.022***	-0.091***	-0.005***	0.001***	-0.007***	-0.035***	-0.016***	**-0.861*****	-0.102***	0.014***	0.000
Vegetables	-0.051***	0.000	-0.007***	-0.091***	-0.023***	-0.004***	0.004***	-0.042***	-0.015***	-0.022***	**-0.749*****	-0.030***	0.101***
Fruits	0.018***	-0.002***	0.006***	-0.074***	-0.021***	0.004***	0.002***	-0.010***	0.012***	0.024***	-0.059***	**-0.892*****	0.043***
Other grains	1.842***	0.281***	0.269***	0.228***	-1.790***	-0.649***	-0.080***	1.308***	-2.162***	0.030***	1.245***	0.301***	**-1.563*****
Conditional expenditure elasticity	0.912***	0.926***	1.028***	1.005***	1.403***	1.296***	1.012***	0.818***	1.058***	1.163***	0.930***	0.949***	0.740***

Notes: * p < 0.10 , *** p < 0.01. The bold font is own-price elasticity.

Table S2. Conditional Hicksian price elasticity (full sample)

	**Rice**	**Wheat**	**Oils**	**Pork**	**Beef**	**Mutton**	**Poultry**	**Eggs**	**Diary**	**Seafood**	**Vegetables**	**Fruits**	**Other grains**
Rice	**-1.005** ***	0.028***	0.051 ***	0.166 ***	0.027 ***	0.039 ***	0.095 ***	-0.047 ***	0.052 ***	0.021 ***	0.046 ***	0.091 ***	0.437 ***
Wheat	0.072 ***	**-1.173*** ***	0.029 ***	0.195 ***	0.068 ***	0.055 ***	0.136 ***	-0.049 ***	0.046 ***	0.064 ***	0.276 ***	0.081 ***	0.199 ***
Oils	0.031 ***	0.013***	**-0.895** ***	0.210 ***	0.026 ***	0.026 ***	0.101 ***	0.022 ***	0.082 ***	0.059 ***	0.152 ***	0.104 ***	0.069 ***
Pork	0.090 ***	0.029***	0.072 ***	**-0.600** ***	0.031 ***	0.000	0.099 ***	-0.002 ***	0.056 ***	0.049 ***	0.089 ***	0.058 ***	0.028 ***
Beef	0.294 ***	0.035***	0.114 ***	0.261 ***	**-0.746** ***	0.011 ***	0.179 ***	0.115 ***	0.061 ***	0.146 ***	0.284 ***	0.145 ***	-0.898 ***
Mutton	0.059 ***	0.041***	0.123 ***	0.057 ***	0.039 ***	**-0.749** ***	0.152 ***	0.185 ***	0.059 ***	0.133 ***	0.220 ***	0.158 ***	-0.479 ***
Poultry	0.076 ***	0.033***	0.069 ***	0.197 ***	0.046 ***	0.027 ***	**-0.898** ***	0.038 ***	0.063 ***	0.075 ***	0.178 ***	0.096 ***	-0.001 ***
Eggs	-0.068 ***	0.020***	0.027 ***	-0.007 ***	-0.018 ***	0.007 ***	0.085 ***	**-0.639** ***	0.042 ***	0.027 ***	0.001	0.072 ***	0.450 ***
Diary	0.025 ***	0.013***	0.073 ***	0.264 ***	0.017 ***	0.004 ***	0.093 ***	0.225 ***	**-0.781** ***	0.098 ***	0.259 ***	0.155 ***	-0.445 ***
Seafood	0.034 ***	0.029***	0.060 ***	0.145 ***	0.030 ***	0.024 ***	0.112 ***	0.018 ***	0.068 ***	**-0.781** ***	0.112 ***	0.130 ***	0.018 ***
Vegetables	0.010 ***	0.021***	0.058 ***	0.098 ***	0.005 ***	0.014 ***	0.099 ***	0.000	0.053 ***	0.042 ***	**-0.578** ***	0.063 ***	0.115 ***
Fruits	0.081 ***	0.020***	0.073 ***	0.118 ***	0.007 ***	0.024 ***	0.099 ***	0.033 ***	0.081 ***	0.090 ***	0.115 ***	**-0.798** ***	0.057 ***
Other grains	1.890 ***	0.298***	0.321 ***	0.378 ***	-1.768 ***	-0.634 ***	-0.004 ***	1.341 ***	-2.108 ***	0.081 ***	1.381 ***	0.375 ***	**-1.551** ***

Notes: *** p < 0.01. The bold font is own-price elasticity.

Table S3. Unconditional Marshall price elasticity and income elasticity (low-income group)

	**Rice**	**Wheat**	**Oils**	**Pork**	**Beef**	**Mutton**	**Poultry**	**Eggs**	**Diary**	**Seafood**	**Vegetables**	**Fruits**	**Other grains**
Unconditional price elasticity													
Rice	**-1.152** ***	0.010***	-0.012 ***	0.052 ***	-0.014 ***	0.011 ***	0.023 ***	-0.135 ***	0.000 ***	-0.029 ***	-0.074 ***	0.038 ***	0.463 ***
Wheat	0.015 ***	**-1.233** ***	-0.040 ***	0.092 ***	0.054 ***	0.047 ***	0.042 ***	-0.053 ***	-0.004 ***	-0.056 ***	0.204 ***	0.026 ***	0.135 ***
Oils	-0.029 ***	-0.013***	**-0.992** ***	0.064 ***	-0.012 ***	0.003 ***	0.023 ***	-0.022 ***	0.030 ***	-0.010 ***	-0.008 ***	0.028 ***	0.059 ***
Pork	0.060 ***	0.019***	0.024 ***	**-0.826** ***	0.004 ***	-0.030 ***	0.007 ***	-0.039 ***	-0.004 ***	-0.002 ***	-0.025 ***	-0.036 ***	-0.020 ***
Beef	0.253 ***	0.023***	0.032 ***	0.072 ***	**-0.786** ***	-0.018 ***	0.091 ***	0.092 ***	-0.006 ***	0.050 ***	0.113 ***	0.015 ***	-1.136 ***
Mutton	-0.042 ***	0.034***	0.048 ***	-0.253 ***	0.001 ***	**-0.687** ***	0.062 ***	0.154 ***	-0.020 ***	0.077 ***	0.078 ***	0.092 ***	-0.701 ***
Poultry	0.035 ***	0.014***	0.019 ***	0.016 ***	0.023 ***	0.012 ***	**-0.975** ***	0.002 ***	-0.010 ***	0.008 ***	0.020 ***	-0.001 ***	-0.021 ***
Eggs	-0.197 ***	0.005***	-0.044 ***	-0.155 ***	-0.046 ***	-0.018 ***	0.008 ***	**-0.587** ***	-0.026 ***	-0.052 ***	-0.125 ***	-0.001 ***	0.555 ***
Diary	-0.040 ***	-0.010***	0.028 ***	0.148 ***	-0.007 ***	-0.019 ***	-0.011 ***	0.275 ***	**-0.882** ***	0.047 ***	0.140 ***	0.102 ***	-0.676 ***
Seafood	-0.024 ***	-0.025***	-0.017 ***	-0.014 ***	-0.020 ***	0.009 ***	0.011 ***	-0.049 ***	0.000 ***	**-0.862** ***	-0.077 ***	0.042 ***	0.046 ***
Vegetables	-0.035 ***	0.022***	-0.002 ***	-0.024 ***	-0.028 ***	0.000 ***	0.012 ***	-0.034 ***	-0.015 ***	-0.021 ***	**-0.769** ***	-0.031 ***	0.142 ***
Fruits	0.060 ***	0.010***	0.028 ***	-0.085 ***	-0.041 ***	0.014 ***	0.000 ***	-0.002 ***	0.031 ***	0.029 ***	-0.069 ***	**-0.852** ***	0.072 ***
Other grains	1.904 ***	0.221***	0.282 ***	-0.231 ***	-1.781 ***	-0.749 ***	-0.111 ***	1.554 ***	-2.460 ***	0.151 ***	1.517 ***	0.357 ***	**-1.323** ***
Income elasticity	0.694 ***	0.651***	0.743 ***	0.734 ***	1.020 ***	0.979 ***	0.724 ***	0.579 ***	0.766 ***	0.829 ***	0.662 ***	0.678 ***	0.565 ***

Notes: *** $p < 0.01$. The bold font is own-price elasticity.

Table S4. Unconditional Marshall price elasticity and income elasticity (lower-middle-income group)

	**Rice**	**Wheat**	**Oils**	**Pork**	**Beef**	**Mutton**	**Poultry**	**Eggs**	**Diary**	**Seafood**	**Vegetables**	**Fruits**	**Other grains**
Unconditional price elasticity													
Rice	**-1.073** ***	0.013***	-0.006 ***	0.025 ***	-0.010 ***	0.018 ***	0.020 ***	-0.097 ***	-0.002 **	-0.037 ***	-0.084 ***	0.025 ***	0.442 ***
Wheat	0.023 ***	**-1.187*** ***	-0.025 ***	0.065 ***	0.042 ***	0.039 ***	0.051 ***	-0.054 ***	-0.004 ***	-0.008 ***	0.149 ***	0.013 ***	0.139 ***
Oils	-0.022 ***	-0.006***	**-0.966** ***	0.046 ***	-0.010 ***	0.003 ***	0.017 ***	-0.013 ***	0.021 ***	-0.009 ***	0.005 ***	0.025 ***	0.060 ***
Pork	0.045 ***	0.015***	0.016 ***	**-0.777** ***	0.001 ***	-0.026 ***	0.012 ***	-0.038 ***	-0.005 ***	-0.013 ***	-0.049 ***	-0.026 ***	0.009 ***
Beef	0.222 ***	0.010***	0.031 ***	0.045 ***	**-0.811** ***	-0.009 ***	0.066 ***	0.081 ***	-0.019 ***	0.055 ***	0.092 ***	0.029 ***	-0.959 ***
Mutton	-0.014 ***	0.021***	0.041 ***	-0.179 ***	0.008 ***	**-0.774** ***	0.043 ***	0.138 ***	-0.014 ***	0.057 ***	0.046 ***	0.067 ***	-0.504 ***
Poultry	0.028 ***	0.016***	0.013 ***	0.025 ***	0.016 ***	0.008 ***	**-0.981** ***	0.006 ***	-0.003 ***	0.009 ***	0.031 ***	0.011 ***	-0.013 ***
Eggs	-0.129 ***	0.006***	-0.025 ***	-0.146 ***	-0.041 ***	-0.011 ***	0.014 ***	**-0.657** ***	-0.012 ***	-0.032 ***	-0.099 ***	0.009 ***	0.452 ***
Diary	-0.027 ***	-0.006***	0.015 ***	0.100 ***	-0.016 ***	-0.017 ***	-0.002 ***	0.203 ***	**-0.855** ***	0.032 ***	0.116 ***	0.079 ***	-0.484 ***
Seafood	-0.030 ***	0.003***	-0.014 ***	-0.049 ***	-0.011 ***	0.004 ***	0.012 ***	-0.029 ***	-0.004 ***	**-0.845** ***	-0.062 ***	0.039 ***	0.014 ***
Vegetables	-0.036 ***	0.011***	0.003 ***	-0.050 ***	-0.027 ***	-0.004 ***	0.018 ***	-0.027 ***	-0.006 ***	-0.018 ***	**-0.728** ***	-0.014 ***	0.114 ***
Fruits	0.040 ***	0.005***	0.021 ***	-0.054 ***	-0.029 ***	0.008 ***	0.012 ***	0.004 ***	0.025 ***	0.027 ***	-0.029 ***	**-0.864** ***	0.058 ***
Other grains	1.835 ***	0.232***	0.284 ***	0.118 ***	-1.792 ***	-0.698 ***	-0.076 ***	1.387 ***	-2.097 ***	0.057 ***	1.327 ***	0.342 ***	**-1.524** ***
Income elasticity	0.567 ***	0.562***	0.629 ***	0.619 ***	0.865 ***	0.791 ***	0.619 ***	0.499 ***	0.639 ***	0.721 ***	0.567 ***	0.575 ***	0.449 ***

Notes: ** $p < 0.05$, *** $p < 0.01$. The bold font is own-price elasticity.

Table S5. Unconditional Marshall price elasticity and income elasticity (middle-income group)

	**Rice**	**Wheat**	**Oils**	**Pork**	**Beef**	**Mutton**	**Poultry**	**Eggs**	**Diary**	**Seafood**	**Vegetables**	**Fruits**	**Other grains**
Unconditional price elasticity													
Rice	**-1.044** ***	0.012***	-0.002 ***	0.014 ***	-0.005 ***	0.023 ***	0.020 ***	-0.080 ***	-0.004 ***	-0.038 ***	-0.082 ***	0.020 ***	0.440 ***
Wheat	0.022 ***	**-1.174*** ***	-0.019 ***	0.051 ***	0.039 ***	0.037 ***	0.055 ***	-0.058 ***	-0.004 ***	0.009 ***	0.126 ***	0.010 ***	0.155 ***
Oils	-0.019 ***	-0.004***	**-0.953** ***	0.041 ***	-0.008 ***	0.004 ***	0.015 ***	-0.009 ***	0.020 ***	-0.005 ***	0.011 ***	0.025 ***	0.059 ***
Pork	0.040 ***	0.013***	0.013 ***	**-0.761** ***	-0.001 ***	-0.020 ***	0.016 ***	-0.033 ***	-0.005 ***	-0.015 ***	-0.053 ***	-0.019 ***	0.021 ***
Beef	0.222 ***	0.008***	0.033 ***	0.034 ***	**-0.805** ***	-0.012 ***	0.061 ***	0.075 ***	-0.023 ***	0.058 ***	0.094 ***	0.040 ***	-0.912 ***
Mutton	0.000 ***	0.017***	0.042 ***	-0.132 ***	0.003 ***	**-0.805** ***	0.041 ***	0.133 ***	-0.014 ***	0.047 ***	0.044 ***	0.054 ***	-0.439 ***
Poultry	0.027 ***	0.016***	0.011 ***	0.032 ***	0.014 ***	0.007 ***	**-0.983** ***	0.007 ***	0.002 ***	0.011 ***	0.039 ***	0.019 ***	-0.011 ***
Eggs	-0.106 ***	0.008***	-0.018 ***	-0.134 ***	-0.042 ***	-0.008 ***	0.016 ***	**-0.677** ***	-0.006 ***	-0.024 ***	-0.101 ***	0.011 ***	0.422 ***
Diary	-0.024 ***	-0.003***	0.012 ***	0.086 ***	-0.018 ***	-0.017 ***	0.005 ***	0.180 ***	**-0.846** ***	0.028 ***	0.109 ***	0.071 ***	-0.422 ***
Seafood	-0.025 ***	0.011***	-0.007 ***	-0.050 ***	-0.008 ***	0.001 ***	0.015 ***	-0.019 ***	-0.004 ***	**-0.847** ***	-0.051 ***	0.041 ***	0.007 ***
Vegetables	-0.034 ***	0.006***	0.005 ***	-0.055 ***	-0.026 ***	-0.004 ***	0.021 ***	-0.026 ***	-0.002 **	-0.016 ***	**-0.710** ***	-0.008 ***	0.103 ***
Fruits	0.033 ***	0.004***	0.019 ***	-0.037 ***	-0.024 ***	0.005 ***	0.019 ***	0.005 ***	0.023 ***	0.029 ***	-0.015 ***	**-0.869** ***	0.050 ***
Other grains	1.856 ***	0.256***	0.281 ***	0.278 ***	-1.838 ***	-0.671 ***	-0.065 ***	1.322 ***	-2.042 ***	0.034 ***	1.269 ***	0.330 ***	**-1.588** ***
Income elasticity	0.476 ***	0.492***	0.540 ***	0.529 ***	0.741 ***	0.663 ***	0.532 ***	0.434 ***	0.552 ***	0.616 ***	0.491 ***	0.499 ***	0.380 ***

Notes: ** $p < 0.05$, *** $p < 0.01$. The bold font is own-price elasticity.

Table S6. Unconditional Marshall price elasticity and income elasticity (upper-middle-income group)

	**Rice**	**Wheat**	**Oils**	**Pork**	**Beef**	**Mutton**	**Poultry**	**Eggs**	**Diary**	**Seafood**	**Vegetables**	**Fruits**	**Other grains**
Unconditional price elasticity													
Rice	**-1.011** ***	0.012***	0.002 ***	0.001 **	0.003 ***	0.027 ***	0.020 ***	-0.055 ***	-0.005 ***	-0.036 ***	-0.077 ***	0.015 ***	0.424 ***
Wheat	0.027 ***	**-1.170*** **	-0.016 ***	0.024 ***	0.037 ***	0.034 ***	0.066 ***	-0.080 ***	-0.007 ***	0.030 ***	0.111 ***	0.006 ***	0.201 ***
Oils	-0.016 ***	-0.001**	**-0.938** ***	0.033 ***	-0.005 ***	0.008 ***	0.015 ***	-0.004 ***	0.019 ***	0.000	0.018 ***	0.026 ***	0.058 ***
Pork	0.036 ***	0.010***	0.010 ***	**-0.739** ***	-0.003 ***	-0.016 ***	0.020 ***	-0.029 ***	-0.004 ***	-0.017 ***	-0.057 ***	-0.011 ***	0.033 ***
Beef	0.220 ***	0.007***	0.036 ***	0.020 ***	**-0.798** ***	-0.018 ***	0.058 ***	0.069 ***	-0.025 ***	0.060 ***	0.097 ***	0.052 ***	-0.839 ***
Mutton	0.012 ***	0.014***	0.051 ***	-0.100 ***	-0.005 ***	**-0.817** ***	0.042 ***	0.142 ***	-0.015 ***	0.043 ***	0.036 ***	0.047 ***	-0.411 ***
Poultry	0.026 ***	0.016***	0.009 ***	0.039 ***	0.012 ***	0.006 ***	**-0.982** ***	0.009 ***	0.008 ***	0.015 ***	0.047 ***	0.027 ***	-0.008 ***
Eggs	-0.075 ***	0.007***	-0.011 ***	-0.125 ***	-0.043 ***	-0.005 ***	0.021 ***	**-0.697** ***	0.001 ***	-0.014 ***	-0.105 ***	0.014 ***	0.388 ***
Diary	-0.020 ***	-0.002***	0.009 ***	0.075 ***	-0.019 ***	-0.016 ***	0.012 ***	0.160 ***	**-0.835** ***	0.026 ***	0.103 ***	0.066 ***	-0.370 ***
Seafood	-0.020 ***	0.017***	-0.002 ***	-0.048 ***	-0.005 ***	-0.002 ***	0.020 ***	-0.010 ***	-0.002 ***	**-0.847** ***	-0.039 ***	0.044 ***	0.000 ***
Vegetables	-0.032 ***	-0.001***	0.007 ***	-0.059 ***	-0.023 ***	-0.005 ***	0.026 ***	-0.026 ***	0.001 ***	-0.014 ***	**-0.688** ***	0.000	0.092 ***
Fruits	0.028 ***	0.003***	0.016 ***	-0.021 ***	-0.017 ***	0.002 ***	0.026 ***	0.005 ***	0.022 ***	0.031 ***	0.000	**-0.877** ***	0.041 ***
Other grains	1.809 ***	0.289***	0.264 ***	0.439 ***	-1.809 ***	-0.610 ***	-0.050 ***	1.186 ***	-1.928 ***	0.001 ***	1.155 ***	0.298 ***	**-1.613** ***
Income elasticity	0.366 ***	0.395***	0.423 ***	0.412 ***	0.568 ***	0.515 ***	0.417 ***	0.346 ***	0.436 ***	0.479 ***	0.388 ***	0.397 ***	0.305 ***

Notes: ** $p < 0.05$, *** $p < 0.01$. The bold font is own-price elasticity.

Table S7. Unconditional Marshall price elasticity and income elasticity (high-income group)

	Rice	Wheat	Oils	Pork	Beef	Mutton	Poultry	Eggs	Diary	Seafood	Vegetables	Fruits	Other grains
Unconditional price elasticity													
Rice	**-0.947** ***	0.010***	0.008 ***	-0.002 ***	0.021 ***	0.026 ***	0.025 ***	-0.001 *	-0.002 ***	-0.031 ***	-0.088 ***	0.007 ***	0.351 ***
Wheat	0.067 ***	**-1.161** ***	-0.013 ***	-0.119 ***	-0.003 ***	0.011 ***	0.102 ***	-0.226 ***	-0.065 ***	0.114 ***	0.113 ***	-0.049 ***	0.486 ***
Oils	-0.005 ***	0.006***	**-0.901** ***	0.030 ***	0.002 ***	0.009 ***	0.015 ***	0.004 ***	0.008 ***	0.010 ***	0.035 ***	0.022 ***	0.061 ***
Pork	0.023 ***	0.002***	0.008 ***	**-0.707** ***	-0.003 ***	-0.008 ***	0.034 ***	-0.025 ***	-0.005 ***	-0.015 ***	-0.067 ***	0.003 ***	0.053 ***
Beef	0.209 ***	0.007***	0.038 ***	0.022 ***	**-0.738** ***	-0.027 ***	0.061 ***	0.038 ***	-0.041 ***	0.081 ***	0.062 ***	0.059 ***	-0.669 ***
Mutton	0.038 ***	0.006***	0.068 ***	-0.063 ***	-0.020 ***	**-0.789** ***	0.051 ***	0.170 ***	-0.029 ***	0.049 ***	0.000	0.035 ***	-0.372 ***
Poultry	0.023 ***	0.014***	0.007 ***	0.060 ***	0.010 ***	0.004 ***	**-0.976** ***	0.010 ***	0.015 ***	0.024 ***	0.058 ***	0.040 ***	-0.002 ***
Eggs	-0.003 ***	0.004***	0.003 ***	-0.145 ***	-0.049 ***	0.000	0.032 ***	**-0.722** ***	0.007 ***	0.010 ***	-0.140 ***	0.013 ***	0.363 ***
Diary	-0.022 ***	-0.005***	-0.001 ***	0.068 ***	-0.021 ***	-0.015 ***	0.027 ***	0.151 ***	**-0.810** ***	0.033 ***	0.102 ***	0.057 ***	-0.346 ***
Seafood	-0.014 ***	0.028***	0.006 ***	-0.036 ***	0.007 ***	-0.002 ***	0.031 ***	0.004 ***	0.005 ***	**-0.853** ***	-0.018 ***	0.048 ***	-0.018 ***
Vegetables	-0.038 ***	-0.014***	0.012 ***	-0.081 ***	-0.019 ***	-0.008 ***	0.040 ***	-0.029 ***	0.004 ***	-0.009 ***	**-0.631** ***	0.012 ***	0.074 ***
Fruits	0.017 ***	-0.003***	0.011 ***	0.006 ***	-0.007 ***	-0.002 ***	0.042 ***	0.004 ***	0.016 ***	0.038 ***	0.018 ***	**-0.885** ***	0.026 ***
Other grains	1.780 ***	0.468***	0.274 ***	0.873 ***	-1.583 ***	-0.428 ***	-0.015 ***	1.042 ***	-2.113 ***	-0.127 ***	1.015 ***	0.229 ***	**-2.023** ***
Income elasticity	0.166 ***	0.198***	0.187 ***	0.188 ***	0.239 ***	0.229 ***	0.190 ***	0.167 ***	0.209 ***	0.216 ***	0.183 ***	0.192 ***	0.162 ***

Notes: * $p < 0.10$, *** $p < 0.01$. The bold font is own-price elasticity.
